# Effect of steam explosion pretreatment on the composition and bioactive characteristic of phenolic compounds in *Chrysanthemum morifolium* Ramat cv. Hangbaiju powder with various sieve fractions

**DOI:** 10.1002/fsn3.2805

**Published:** 2022-03-15

**Authors:** Gongshuai Song, Jiayuan Liu, Ruofan Shui, Jiachen Sun, Qian Weng, Shaoping Qiu, Danli Wang, Shiwang Liu, Gongnian Xiao, Xi Chen, Qing Shen, Jinyan Gong, Fuping Zheng

**Affiliations:** ^1^ 91616 Zhejiang Provincial Key Lab for Biological and Chemical Processing Technologies of Farm Product School of Biological and Chemical Engineering Zhejiang University of Science and Technology Hangzhou China; ^2^ Beijing Laboratory of Food Quality and Safety Beijing Technology and Business University Beijing China; ^3^ Zhejiang Provincial People’s Hospital Affiliated People’s Hospital of Hangzhou Medical College Hangzhou China; ^4^ Collaborative Innovation Center of Seafood Deep Processing Zhejiang Province Joint Key Laboratory of Aquatic Products Processing Institute of Seafood Zhejiang Gongshang University Hangzhou China

**Keywords:** Hangbaiju powder, in vitro digestion property, phenolic compound, sieve fraction, steam explosion

## Abstract

Steam explosion (SE) pretreatment is an efficient technique to promote the fiber degradation and disrupt materials' cell wall. In this study, the effect of SE pretreatment on the changes in phenolic profile, and the in vitro digestion property of a Chinese indigenous herb “Hangbaiju” (HBJ) powder with various sieve fractions (150‐, 180‐, 250‐, 425‐, and 850‐μm sieves) were studied. After SE pretreatment, the morphological structure, color attributes, and composition of phenolic compounds were altered significantly (*p* < .05). The composition and content of phenolic compounds were strongly correlated with particle sizes. The higher extraction yield of phenolic compounds was reached in the intermediate sieve fraction (ca. 250‐μm sieves). During in vitro digestion, the changes in phenolic compounds were significant due to the transition from an acidic to the alkaline environment (*p* < .05). Based on the multivariate statistical analysis, apigenin‐7‐O‐glucoside, luteolin‐7‐O‐glucoside, and linarin, were viewed as the characteristic compounds among various samples. The results highlighted that the phytochemical properties mainly including the composition of phenolic compounds, and in vitro digestion properties of HBJ powder with intermediate sieve fraction could be improved after SE pretreatment.

## Graphical abstract

The color attributes and morphological structure of HBJ powders were altered by SE pretreatment. The content of phenolic compounds, and in vitro digestion properties were related to particle sizes. The superior phytochemical properties were reached in intermediate size (ca. 250 μm sieves).

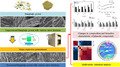

## 1 INTRODUCTION

*Chrysanthemum morifolium* belonging to the Asteraceae family has been widely cultivated for more than 3000 years in Zhejiang Province, China, which is an edible and medicinal cognate. Its flowers are usually used as a herbal medicine to treat skin itch, dim eyesight, common cold, and dizziness in Traditional Chinese Medicine (TCM) (Kuang et al., 2018; Xie et al., 2012). “Hangbaiju” (HBJ) is one of the kinds of *Chrysanthemum morifolium* Ramat, which is viewed as the classical tea material and key herbal medicine (Gong et al., 2021). Many biological and pharmacological effects of HBJ have been reported, such as anticarcinogenic, anti‐inflammatory, antimutagenic activity, cardiovascular protection, radical‐scavenging, and antioxidant (He et al., 2021; Lin & Harnly, 2010). It was found that HBJ contained significant amounts of biologically active components contributing to the health benefits, mainly including polysaccharides, flavonoids (e.g., apigenin and luteolin), triterpenes, and caffeoylquinic acid (CQA) (Gong et al., 2019). According to the number or the position of caffeoyl groups, CQAs are classified into various derivatives. Skala et al. (2020) demonstrated that mono‐CQAs are the most abundant (Skala et al., 2020). It was worth extracting and determining these chemical compounds for expanding the HBJ application. However, the traditional extraction methods are environmentally harmful and time‐consuming. It is essential to explore an alternative processing technique.

Steam explosion (SE) is an innovative and economical technique that has been widely applied for the pretreatment process in many fields (e.g., bioactive phytochemical extraction) with time‐saving, low cost, and pollution (Gong et al., 2021). During SE pretreatment, raw samples are processed under a temperature that ranged from 160°C to 260°C, with the corresponding pressure ranging from 0.69 to 4.83 MPa. The steam enters the internal space of raw materials, followed by a sudden decompression for physical treatment of the samples (Cui et al., 2021). After SE pretreatment, the cell volume is enlarged and the cell wall is damaged, which accelerates the release of low‐molecular‐weight substances from cells (Gong et al., 2012). Chen et al. (2020) used the SE pretreatment to extract valuable phytochemicals from soybean seed coats, and the result revealed that the phenolic profiles and antioxidant activity of soybean seed coats were enhanced after SE pretreatment (Chen et al., 2020). Romero‐García et al. (2016) reported that SE pretreatment was successfully applied as a procedure to extract natural antioxidants and sugars from olive tree leaves (Romero‐García et al., 2016). Chen et al. (2011) found that SE could reduce the particle size and form large fissures and micropores on the sumac fruit coat, and the extraction yield of flavonoids from samples treated by SE was ca. 8 times higher than that from raw materials (Chen & Chen, 2011). Furthermore, it has been proved that SE pretreatment was an effective method for extracting phenolic compounds from samples (Gong et al., 2012).

Recently, HBJ has been widely consumed as healthy foods (herb tea alone) or in combination with other active substances such as wolfberry tea because of the remarkable antioxidant capacity (Zhang et al., 2019). Moreover, in vitro simulated digestion of food ingredients has attracted much attention in the field of food and nutrition sciences (Wang et al., 2019). It was reported that the absorption of phenolic acids from an edible matrix is easily affected by the acidic pH value during gastrointestinal (GI) digestion, and only a small part of phenolic acids is available for exerting the antioxidant activity (de Morais et al., 2020). It is essential to consider the change in phenolic profile of HBJ during digestion. However, whether the effect of SE pretreatment on HBJ with various particle sizes can change the composition of phenolic compounds, and the digestion property is still unknown. The aim of this study was to evaluate the effect of SE pretreatment on phenolic profiles (3‐CQA, 4‐CQA, 5‐CQA, 3,4‐CQA, 3,5‐CQA, 4,5‐CQA, linarin, acacetin, apigenin, luteolin, apigenin‐7‐O‐glucoside, luteolin‐7‐O‐glucoside), and in vitro simulated digestion properties of HBJ with various particle sizes.

## 2 MATERIALS AND METHOD

### 2.1 Materials

The dried HBJ flowers (300 g, moisture content was ca. 10%) were harvested from Tongxiang, Zhejiang province, China. The obtained samples were ground into powders, which were selected and divided into five sieve fractions (850, 425, 250, 180, and 150 μm corresponding to 20, 40, 60, 80, and 100 meshes, respectively). The representative external standards with the purity of 98% were all provided by Chengdu Must Bio‐Technology Co. Ltd., namely, 3‐CQA, 4‐CQA, 5‐CQA, 3,4‐CQA, 3,5‐CQA, 4,5‐CQA, linarin, apigenin, acacetin, rutin, luteolin, apigenin‐7‐O‐glucoside, and luteolin‐7‐O‐glucoside. Acetonitrile (ACN) and methanol were purchased from Merck of chromatographic grade. Hydrochloric acid (HCl), ethyl acetate, acetone, potassium peroxodisulfate (K_2_S_2_O_8_), ethyl alcohol, acetic acid, monopotassium phosphate (KH_2_PO_4_), disodium hydrogen phosphate (Na_2_HPO_4_), and ferric chloride with analytical grade were bought from Sinopharm Chemical Reagent Co., Ltd. The ultrapure water (18.2 MΩ•cm) was produced using a Milli‐Q system (Millipore). α‐Amylase, pepsin from porcine gastric mucosa (enzymatic activity of 250 U/mg protein), and pancreatin from porcine pancreas (enzymatic activity of 24 U/mg of lipase) were purchased from Sigma Chemical Co.

### 2.2 SE pretreatment

According to the method described in Gong et al. (2021) with slight modifications, the SE pretreatment of HBJ samples was carried out (Gong et al., 2021). Briefly, the dried sample was pretreated in the SE jar with a piston (Henan, China). In the SE jar, the high‐temperature and high‐pressure water steam was generated and entered into a cylinder by an air valve. Afterwards, the inlet air valve was opened and the pressure was released simultaneously to produce the SE on the samples. Before the explosion, the jar was kept at 1.5 MPa for 90 s. Then the samples were exploded for 150 s. After SE pretreatment, the samples were collected and then dried under vacuum freeze (Beta 2–8 LD plus, Christ, Germany), and stored at −20°C for further analysis.

### 2.3 Scanning electron microscope (*SEM*)

The morphology of HBJ powders with various sieve fractions was performed using a scanning electron microscope (*SEM*) (FEI Inspect F50, Thermo Fisher Scientific) at an accelerating voltage of 1.2 kV. The powders were equally distributed on conductive adhesive tape and coated with gold–palladium alloy. The micrographs were observed under a low vacuum.

### 2.4 Color analysis

The surface color of the HBJ samples treated with SE pretreatment with various sieve fractions was analyzed with a spectrophotometer (HunterLab). Before the experiment, the apparatus was adjusted to blank with white calibration plate. The parameters of L* (lightness, L* = 0 black and L* = 100 white), a* (green–redness chromacity, − a* = greenness and +a* = redness), and b* (green−red chromacity, − b* = blueness and +b* = yellowness) were measured for five technical replicates to present the color of the samples. The powder without SE pretreatment was used as the reference.

### 2.5 Sample extraction

The extraction procedure of HBJ samples with various sieve fractions before and after SE pretreatment was conducted according to the previous method reported by Liu et al. (2016) with slight modifications (Liu et al., 2016). In brief, the samples (0.5 g) were mixed with 70% (v/v) methanol water solution with sonication under 40 kHz at 50°C for 40 min. After centrifuged with 8 000 g for 15 min, the supernatants were combined and filtered through 0.45‐μm polytetrafluoroethylene (PTFE) membrane for further analysis.

### 2.6 Determination of the total phenolic content

According to the reported method with slight modification, the total phenol contents of samples were determined using chlorogenic acid as the external standard (Zhang et al., 2018). Briefly, 1.0 ml of diluted sample solution was mixed with the Folin–Ciocalteu reagent (1.0 ml) and Na_2_CO_3_ solution (1 M, 5.0 ml). Then the mixture was blended with Milli‐Q water (3 ml), which was placed in darkness at room temperature for 2 hr. The absorbance was read at 760 nm on a UV‐5200PC spectrophotometer (Metash Instrument). The total phenol contents in the sample were expressed as mg chlorogenic acid equivalents per gram dry weight (DW).

### 2.7 Determination of the total flavonoid content

According to the reported method with slight modification, the total flavonoid contents of samples were determined by the NaNO_2_−Al(NO_3_)_3_ colorimetric method using rutin as the external standard (Chen et al., 2020). In brief, 1.0 ml of diluted sample solution was mixed with Milli‐Q water (4.0 ml) and NaNO_2_ solution (5%, w/v, 0.3 ml) at room temperature for 5 min. Afterwards, the above solution was mixed with Al(NO_3_)_3_ solution (10%, w/v, 0.3 ml) for 6 min under continuous magnetic stirring. Then NaOH solution (1 M, 2 ml) was added into the mixture and brought to the final volume at 10 ml with 30% ethanol. The absorbance of the mixture was read at 510 nm. The total flavonoid content in these samples was expressed as milligram rutin equivalents per gram DW.

### 2.8 High‐performance liquid chromatography (HPLC) analysis

The HPLC analysis of phenolic compounds in the samples was carried out by the modified method of Gong et al. (2021). An Agilent 1260 series HPLC coupled with a Phenomenex Luna C18 column (250 nm ◊ 4.6 mm, 5 μm, Torrance, CA, USA) was applied for the qualitative and quantitative analysis (Agilent Technologies). The binary gradient mixtures including acetonitrile (ACN) (mobile phase A) and 0.1% (v/v) phosphoric acid in water (mobile phase B) were used as the mobile phase at a flow rate of 0.8 ml/min. The column was maintained at 35°C and the injection volume was 10 μl. In this study, the superior wavelength was chosen as 330 nm. By comparing the corresponding external standard, the phenolic compounds were identified and quantified. Gradient elution was performed as given in Table S1.

### 2.9 In vitro gastric–intestinal digestion

According to the standardized INFOGEST protocol described in Minekus et al. (2014) with slight modifications, the in vitro gastric–intestinal digestion procedures were carried out (Minekus et al., 2014). By maintaining the physiological compositions of enzymes, pH, and electrolytes, the simulated gastric fluid (SGF) and simulated intestinal fluid (SIF) were prepared (Liu et al., 2019; Liu et al., 2020). The composition of simulated GI digestion juices is shown in Table 1. In brief, the samples were blended with SGF at a ratio of 1:1 (w/v) and properly homogenized. The pH of the mixture was adjusted to 3 with HCl (1 M). Then the mixture was placed in the incubator chamber for 120 min at 37°C under continuous magnetic stirring. Finally, the SIF was added and the pH was adjusted to 7.0 with NaOH (1 M) at a volume ratio of 1:1. Then the mixture was placed in the incubator chamber at 37°C for 120 min under continuous magnetic stirring. After the enzymatic reactions were halted, the digestive juice was dried under vacuum freeze and stored at −20°C for further analysis.

**TABLE 1:** Compositions of simulated gastrointestinal (GI) digestion juices

Compounds	Concentration (mM)	
	SGF (pH = 3)	SIF (pH = 7)
KH_2_PO_4_	0.90	0.8
KCl	6.90	6.80
NaCl	47.20	47.20
NaHCO_3_	25.00	85.00
CaCl_2_	0.15	0.60
(NH_4_)_2_CO_3_	0.50	−
MgCl_2_·(H_2_O)_6_	0.10	0.33
Pancrelipase	2000 U/ml	2000 U/ml
Trypsin	−	100 U/ml
Pepsin	2000 U/ml	−
Bile salt	0.2	20

Abbreviations: SGF, simulated gastric fluid; SIF, simulated intestinal fluid.

### 2.10 Statistical analysis

Using Microsoft office software, Origin 8.0, and IBM SPSS Statistics (version 21.0), the experimental data were processed. The results were expressed in table form as mean ±standard deviation. Statistical analyses were performed by the analysis of variance (ANOVA). Each experiment's measurement was carried out in triplicate. The correlation was inspected by the Pearson correlation analysis (2‐tailed) and visualized by R version 3.6.2. In order to have an overview of all the samples, a principal component analysis (PCA) was performed. The variability present in the obtained dataset was performed by MetaboAnalyst 5.0 (McGill University, Montreal, Canada).

## 3 RESULTS

### 3.1 Morphological changes

**FIGURE 1:**
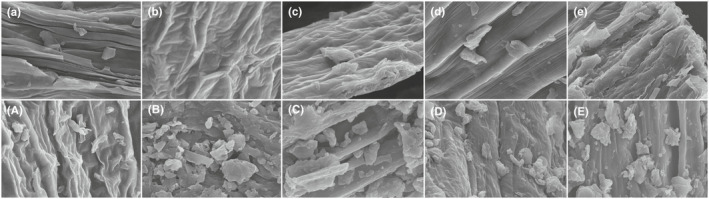
Scanning electron microscopy (*SEM*) images of various Hangbaiju (HBJ) powder samples. (a: Unexploded 20‐mesh; A: exploded 20‐mesh; b: unexploded 40‐mesh; B: exploded 40‐mesh; c: unexploded 60‐mesh; C: exploded 60‐mesh; d: unexploded 80‐mesh; D: exploded 80‐mesh; e: unexploded 100‐mesh; E: exploded 100‐mesh)

The *SEM* images are shown in Figure 1, and the effect of the SE pretreatment on HBJ samples with various sieve fractions' (20, 40, 60, 80, and 100 meshes) surface structure was observed. It could be seen that the texture of the unexploded 20‐mesh sample (Figure 1a) was clear, the veins were neat, and the cell wall structure was relatively complete. After SE pretreatment, the surface of HBJ sample (Figure 1a) became compact, the texture tended to be rough, and the cell wall ruptured, which caused the formation of large cavities and fragments. With the increase in the number of meshes, the structure was ruptured totally (Figure 1b,c,d,e). Besides, the fragmented HBJ sample treated by SE was closely linked together and the viscosity increased. It has been demonstrated that the plant with a cellular structure can produce some acidic substances after high‐temperature process under certain pressure and temperature medium. Parts of the hemicellulose in the plant will degrade into soluble sugar, which increases the adhesion between cells (Li et al., 2007). During SE pretreatment, the saturated water steam seeped into the HBJ samples and a large amount of mechanical energy was formed in an incredibly short time, leading to the cell wall in getting broken. The formed mechanical shearing force acted on the sample directly, resulting in the structural destruction (Wang et al., 2021). The effect of SE pretreatment on HBJ samples with larger sieve fraction was beneficial to the dissolution of active substance.

### 3.2 Color difference

**FIGURE 2:**
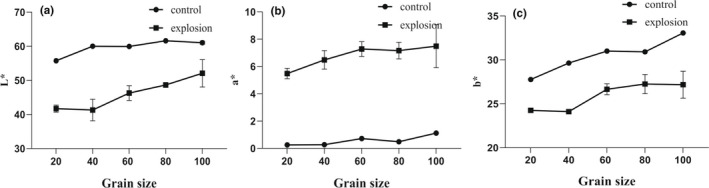
The changes in color difference of Hangbaiju (HBJ) samples before and after steam explosion (SE) pretreatment

Color is one of the most important indexes for evaluating the change in HBJ quality after SE pretreatment. The moisture loss and structural fracture induced by heat and mass transfer are related to the color change of exploded HBJ samples. The variation of L^*^, a^*^, and b^*^ of samples with various sieve fractions before and after SE pretreatment is shown in Figure 2. L^*^ value is viewed as a key parameter defined for the brightness. The L^*^ value of exploded samples was lower than that of unexploded samples, and the value increased with the decrease of particle sizes. A similar change trend could be seen in the b* value of exploded samples. The result indicated that the SE pretreatment possessed a darker appearance and the color of smaller particles was deepener. Compared with unexploded samples, the a* value was higher and increased with the decrease of particle sizes after SE pretreatment, revealing that SE pretreatment can favor the red hue intensity in the samples. It has been reported that the composition of pigment is unstable and gets destroyed under high temperature and high pressure (Gong et al., 2021).

### 3.3 Changes in total flavonoids and total phenols

The change trends of total flavonoids and total phenols in HBJ samples with various sieve fractions are shown in Figure S1. After SE pretreatment, the total phenol content was higher significantly than that in the unexploded samples with the same particle size (*p* < .05), for example, 20‐mesh sample and 40‐mesh sample. However, the difference in the total phenols between exploded and unexploded samples did not become obvious with the decrease in particle size. The total flavonoid content increased with the increase in mesh number after SE. The total phenol content in an exploded 20‐mesh sample was only 48.95 mg of chlorogenic acid equivalents/g DW, the highest total phenol content was obtained in exploded 60‐mesh sample (48.95 mg/g). In addition, for unexploded samples, the total flavonoid content was higher than that in exploded sample with the sample particle sizes. With the decrease of particle sizes, the content of flavonoids increased. But the difference among 60‐mesh, 80‐mesh, and 100‐mesh samples was insignificant (*p* > .05).

### 3.4 Changes in representative phenols and flavonoids

**FIGURE 3:**
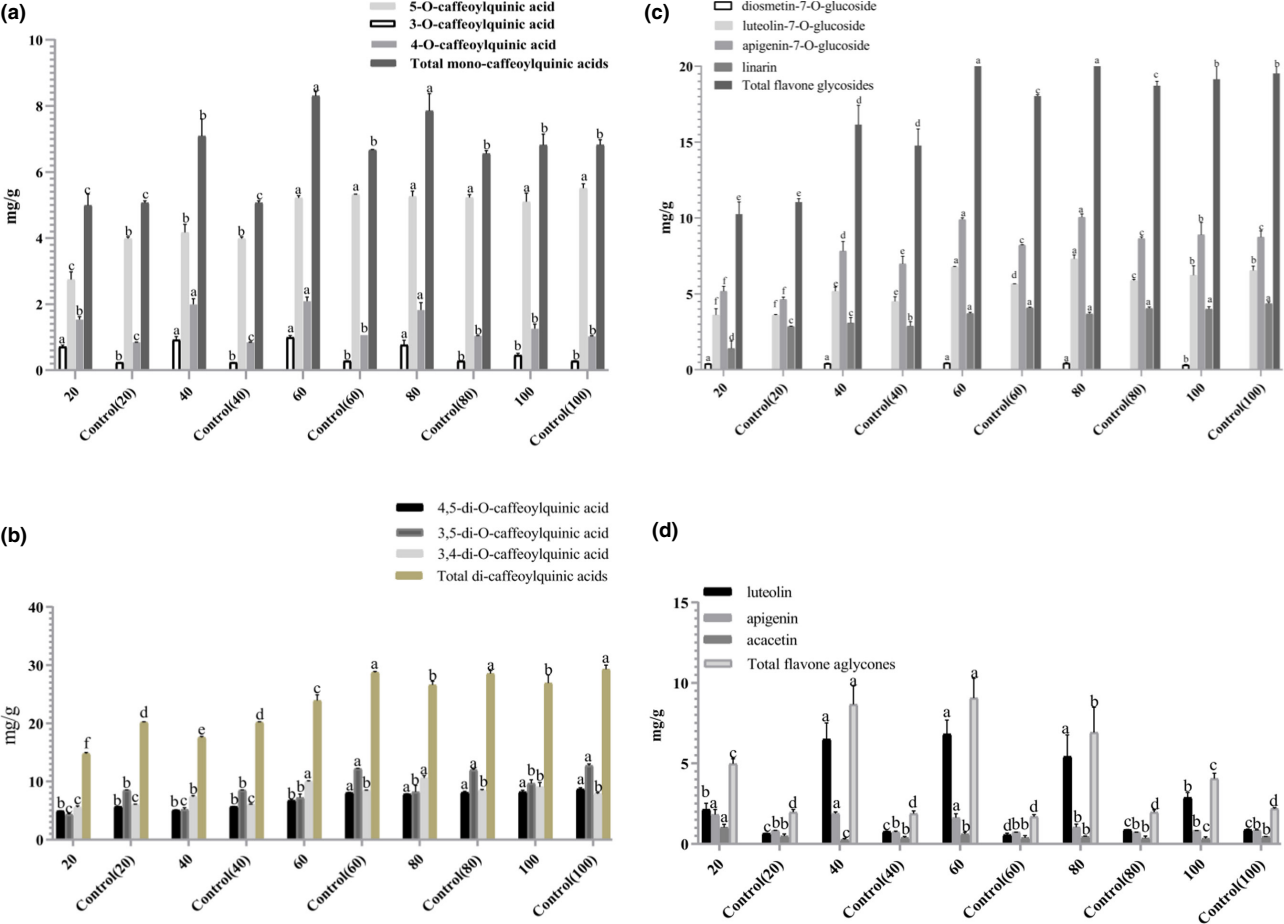
Effects of steam explosion (SE) pretreatment on mono‐CQA (caffeoylquinic acid) contents (a), di‐CQA contents (b), flavone glycoside contents (c), and flavone aglycone contents (d) in Hangbaiju (HBJ) samples with various sieve fractions (20, 40, 60, 80, and 100 meshes), respectively. Different letters indicate a significant difference (*p* < .05)

In this study, the changes in representative phenols and flavonoids of HBJ samples with various sieve fractions were analyzed before and after SE pretreatment. Three mono‐CQA substances (3‐CQA, 4‐CQA, and 5‐CQA) and three di‐CQA substances (3,4‐CQA, 3,5‐CQA, and 4,5‐CQA) were chosen as the representative phenols. As shown in Figure 3a, the contents of 3‐CQA, 4‐CQA, and 5‐CQA in exploded samples increased first and then decreased with the decrease of particle sizes. The highest content was obtained in the 60‐mesh sample. Compared with unexploded samples with same particle size, the 3‐CQA and 4‐CQA contents increased significantly (*p* < .05), whereas the 5‐CQA content decreased after SE pretreatment. As depicted in Figure 3b, the contents of 3,5‐CQA and 4,5‐CQA in exploded samples constantly increased with the decrease of particle sizes. The 3,5‐CQA content was significantly lower than that of unexploded sample with the same particle size. The 3,4‐CQA content increased first and then decreased, and reached the highest value of 10.57 mg/g in a 60‐mesh sample, which was higher than that of the unexploded sample.

The contents of four flavonoid glycosides (diosmetin‐7‐O‐glucoside, luteolin‐7‐O‐glucoside, apigenin‐7‐O‐glucoside, and linarin) are shown in Figure 3c. In the unexploded samples, the contents of luteolin‐7‐O‐glucoside, apigenin‐7‐O‐glucoside, and linarin increased with the decrease of particle sizes. After SE pretreatment, the contents of diosmetin‐7‐O‐glucoside, luteolin‐7‐O‐glucoside, and apigenin‐7‐O‐glucoside increased first and then decreased with the decrease of particle sizes, which were higher than that of unexploded samples. It was noted that diosmetin‐7‐O‐glucoside could be detected after SE pretreatment, the highest content was obtained in the 60‐mesh sample (0.46 mg/g). As shown in Figure 3d, the contents of luteolin and apigenin increased first and then decreased, whereas the acacetin content decreased with the decrease of particle sizes after SE pretreatment. The highest total flavone aglycone content (9.07 mg/g) was obtained in the exploded 60‐mesh sample, which increased by 436.69% compared to the unexploded 60‐mesh sample. The differences in the flavone aglycone contents of the unexploded samples with various sieve fractions were not significant.

### 3.5 Changes in phenolic compounds during in vitro digestion

For evaluating the digestion property of HBJ samples before and after SE pretreatment, the total phenolic compound content was measured by in vitro digestion models. On the basis of the above results, the 20‐mesh, 40‐mesh, and 60‐mesh samples were chosen as the experimental materials. As shown in Figure S2, the total phenol and total flavonoid contents of exploded HBJ samples were higher than the unexploded samples with the same particle size after in vitro simulated GI tract. Furthermore, the content increased with the decrease of particle sizes. After SE pretreatment, the highest total phenol and total flavonoid contents were obtained in 60‐mesh HBJ samples after the gastric phase of in vitro digestion, which were 45.95 mg/g and 39.41 mg/g, respectively. Additionally, the highest total phenol and total flavonoid contents were also obtained in 60‐mesh HBJ samples after the intestinal phase of in vitro digestion, which were 41.27 mg/g and 25.35 mg/g, respectively. The contents of total flavonoids and total phenols decreased significantly during in vitro digestion (*p* < .05). The result demonstrated that the phenols and flavonoids could be degraded by SGF and SIF, and the extraction yield of phenols and flavonoids in 60‐mesh samples was higher after SE pretreatment.

**FIGURE 4:**
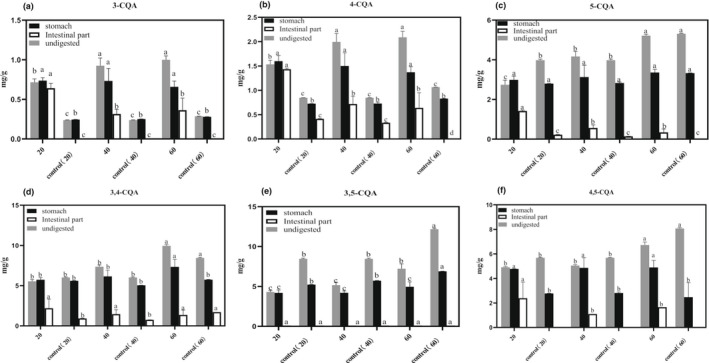
The changes in mono‐CQA (caffeoylquinic acid) contents (a: 3‐CQA; b: 4‐CQA; c: 5‐CQA) and di‐CQA contents (d: 3,4‐CQA; e: 3,5‐CQA; f: 4,5‐CQA) of Hangbaiju (HBJ) samples with various sieve fractions (20, 40, and 60 meshes) before and after steam explosion (SE) pretreatment during in vitro digestion. Different letters indicate a significant difference (*p* < .05)

For further analysis of the changes in the contents of characteristic phenols and flavonoids during in vitro digestion, 3‐CQA, 4‐CQA, 5‐CQA, 3,4‐CQA, 3,5‐CQA, 4,5‐CQA, luteolin‐7‐O‐glucoside, apigenin‐7‐O‐glucoside, linarin, luteolin, apigenin, and acacetin were chosen as the representative compounds. As depicted in Figure 4a–c, the contents of three mono‐CQAs (3‐CQA, 4‐CQA, and 5‐CQA) were all higher than unexploded samples with same particle sizes after SE pretreatment during in vitro digestion (*p* < .05). Compared with the contents of mono‐CQAs after the gastric phase of in vitro digestion, the mono‐CQA contents reduced much more after the intestinal phase of in vitro digestion (*p* < .05). The effects of SE pretreatment on di‐CQA contents with various particle sizes during in vitro digestion were evaluated (Figure 4d–f). During in vitro digestion, the contents of 3,4‐CQA and 4,5‐CQA in exploded samples were both higher than unexploded samples with same particle sizes, whereas 3,5‐CQA content was lower (*p* < .05). With the decrease in particle sizes, the 3,4‐CQA and 4,5‐CQA contents in the exploded samples decreased. However, the 3,5‐CQA was not detected in the samples after in vitro digestion.

**FIGURE 5:**
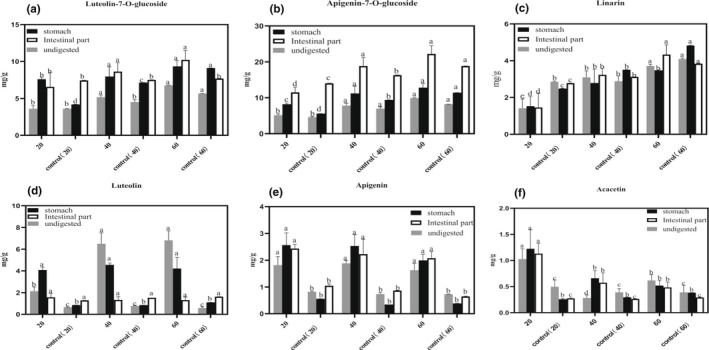
The changes in flavone glycoside contents (a: luteolin‐7‐O‐glucoside; b: apigenin‐7‐O‐glucoside; c: linarin) and flavone aglycone contents (d: luteolin; e: apigenin; f: acacetin) of Hangbaiju (HBJ) samples with various sieve fractions (20, 40, and 60 mesh) before and after steam explosion (SE) pretreatment during in vitro digestion. Different letters indicate a significant difference (*p* < .05)

As depicted in Figure 5a–c, the contents of luteolin‐7‐O‐glucoside, apigenin‐7‐O‐glucoside, and linarin in exploded samples were all higher than unexploded samples with same particle sizes after in vitro digestion. With the decrease of particle sizes, the three flavonoid glycoside contents in the exploded samples increased. The contents of digested flavonoid glycosides were higher than that of the undigested. The effects of SE pretreatment on the contents of flavonoid aglycones (luteolin, apigenin, and acacetin) with various particle sizes during in vitro digestion were carried out (Figure 5d–f). Compared with unexploded samples with the same particle sizes, the contents of luteolin, apigenin, and acacetin in exploded samples increased after in vitro digestion. With the decrease of particle sizes, the contents of flavonoid aglycones increased.

### 3.6 Multivariate statistical analysis

**FIGURE 6:**
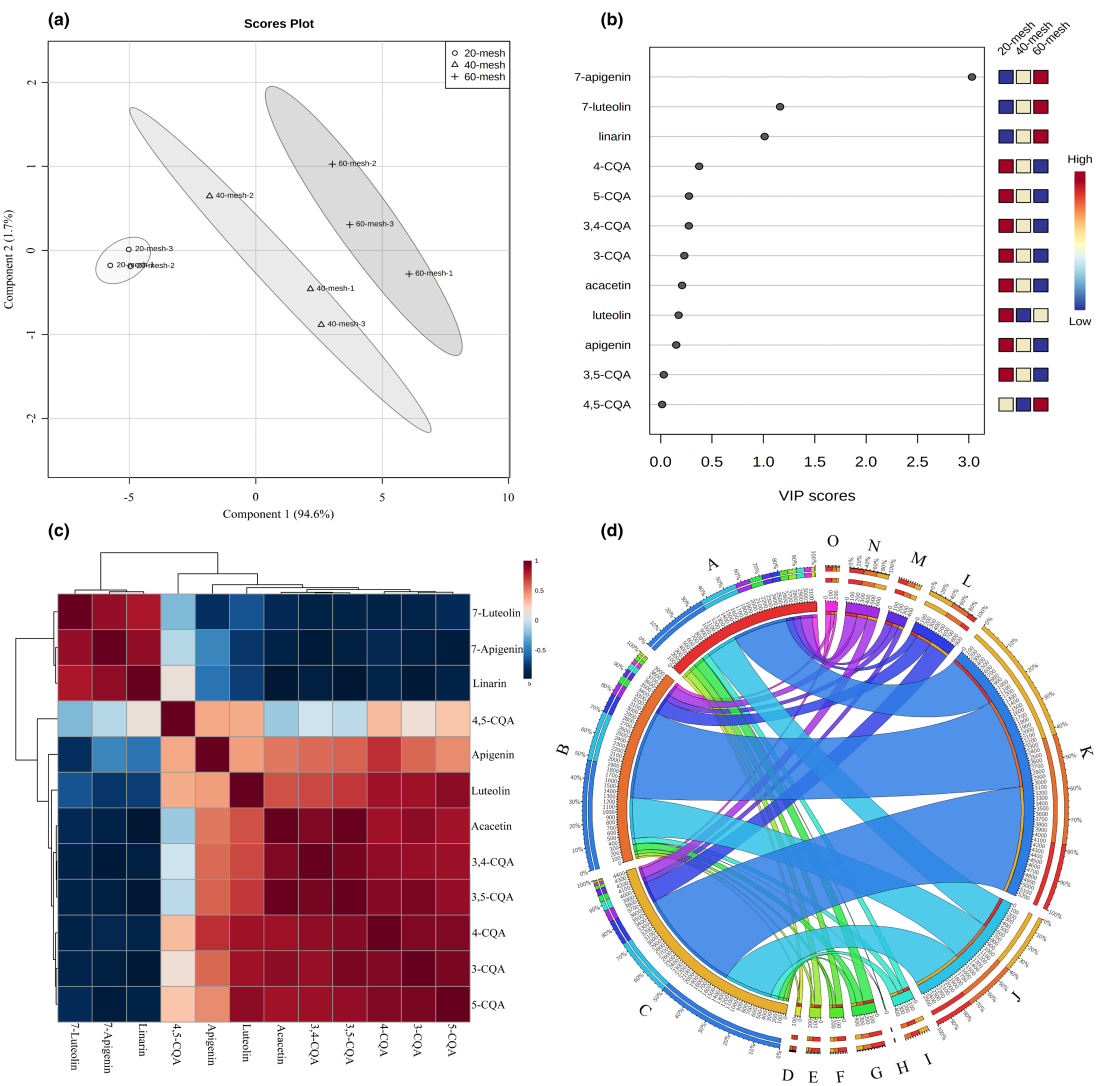
Multivariate statistical analysis of phenolic compounds from various samples. (a) Score plot of the principal component analysis (PCA). (b) Variable importance on projection (VIP) plot of partial least squares regression discriminant analysis (PLS‐DA). (c) Correlation heatmap analysis. (d) Circosplot shows an intuitive result of the correlation coefficients between the samples and various phenolic compounds. (A: 20‐mesh; B: 40‐mesh; C: 60‐mesh; D: 3‐CQA; E:4‐CQA; F: 5‐CQA; G: 3,4‐CQA; H: 3,5‐CQA; I: 4,5‐CQA; J: luteolin‐7‐O‐glucoside; K: apigenin‐7‐O‐glucoside; L: linarin; M: luteolin; N: apigenin; O: acacetin)

For analyzing the difference in the phenols (3‐CQA, 4‐CQA, 5‐CQA, 3,4‐CQA, 3,5‐CQA, and 4,5‐CQA) and flavonoids (luteolin‐7‐O‐glucoside, apigenin‐7‐O‐glucoside, linarin, luteolin, apigenin, and acacetin) among 20‐mesh, 40‐mesh, and 60‐mesh samples during in vitro digestion, multivariate statistical analysis was carried out. The unsupervised PCA is a common pattern recognition method, which can reduce the dimensions of large variables and minimize the loss of original data (Song et al., 2019). As the score plot shown in Figure 6a, the three HBJ samples viewed as the uncorrelated principal components (PCs) were distinctively separated into three clusters and the first two PCs explained 96.3 cum % of total variance of the dataset, revealing a good distribution of the samples in space (Saerens et al., 2004). For supervised data analysis, the partial least squares regression discriminant analysis (PLS‐DA) was used, predicting the Y‐variable (samples) based on the X‐matrix (phenols and flavonoids) by building the prediction model (Song, Chen, et al., 2020). The obtained PLS‐DA model indicated good fitness and predictive ability with R^2^(Y) = 0.991 and Q^2^ = 0.979. In addition, variable importance on projection (VIP) analysis is conducted by the algorithms in the PLS‐toolbox, which can reveal the significance of the variable for separating different samples. The variables with the value of VIP >1 were viewed as the criterion for significant variables (Song, Wang, et al., 2021). As shown in Figure 6b, three flavone glycosides (apigenin‐7‐O‐glucoside, luteolin‐7‐O‐glucoside, and linarin) were the potential phenolic compounds with VIP value >1 among three samples. According to the Pearson correlation analysis, the heatmap of correlation was performed for further analysis of correlation coefficients of phenols and flavonoids. As depicted in Figure 6c, the 3,5‐CQA showed a positive correction to 3‐CQA (*r* = .785, *p* < .05) and 3,4‐CQA (*r* = .836, *p* < .05), whereas a negative correlation to linarin (*r* = −.887, *p* < .05). Circos map as a novel visual tool can identify and analyze the similarities and differences of samples, which can reveal the relationships between pairs of positions using the circular ideogram layout. Moreover, using histogram plots, connectors and lines display the correlation of the data by an aesthetic and intuitive way (Song, Wang, et al., 2020; Song, Zhu, et al., 2021). Figure 6d reveals that the strong correlation between the dominant phenols and flavonoids and the samples corresponded to the thickness of lines, indicating that 60‐mesh samples had a significant correlation with the detected phenols and flavonoids.

## 4 DISCUSSION

It has been reported that total phenols in plants could increase after SE pretreatment. Liu et al. (2016) used SE pretreatment technique to release the phenolic acids bound to the cell walls from wheat bran effectively, and the content of individual phenolic acid increased from 55.7 to 586.3 μg/g (Liu et al., 2016). However, it should be noted that the degradation of phenols can be induced by SE due to high temperature (Deshpande et al., 2014). Besides, it was found that part of formed organic acids (e.g., acetic acid, levulinic acid, and formic acid) made the reaction system acidic and promoted the polymerization reaction (Li et al., 2007). In this study, the results revealed that the total phenol contents in the samples with the same particle size increased after SE pretreatment, which was in line with the previous report (Liu et al., 2016). But the total flavonoid content was lower in the exploded samples, which was attributed to the chemical composition destruction of samples processed by the SE pretreatment. For HBJ samples with various sieve fractions, it could be explained that larger particles contained more fibers and less bioactive compounds. Besides, the smaller particles with larger specific surface area were easily subjected to a higher temperature, leading to the decomposition of thermosensitive biomolecules such as phenolic compounds (Becker et al., 2016).

The results demonstrated that SE pretreatment and suitable sieve fraction could enhance the extraction yield of mono‐CQA and di‐CQA substances. Sieving clearly impacted the chemical composition of powders. Smaller particles appeared richer in minerals, lipids, and proteins, but poorer in carbohydrates and moisture (Zaiter et al., 2016). It has been reported that smaller particles may have higher content in bioactive compounds with greater dry matter content, but it may be subject to heating effect easily, which exerted a negative effect on bioactive compounds. Becker et al. (2017) revealed that the intermediate particle size may be the superior sieve fraction for bioactive compound retention (Becker et al., 2017). Our result was in accordance with the report. It has been demonstrated that the contents of 5‐CQA and 3,5‐CQA exhibited a dramatic decrease in coffee beans treated at high temperature. In addition, 5‐CQA and 3,5‐CQA were more likely to be transformed into 3‐CQA and 4‐CQA, while 3.5‐CQA was easier to be degraded into 5‐CQA, 3,4‐CQA, and 4,5‐CQA (Kamiyama et al., 2015).

Generally, flavonoids are commonly found as secondary metabolites in plants. It was found that the heat treatment may destroy the plant cell wall and enhance the release of bound flavonoid and polyphenolic compounds. Furthermore, the synergies mainly including hydrogen bond destruction, quasi‐mechanical fracture, structural rearrangement, and thermal degradation may disrupt the chemical bond between the lignin and phenolics during the SE pretreatment, which could also improve the dissolution rate of flavonoid (Li et al., 2020). Apigenin‐7‐O‐glucoside, luteolin‐7‐O‐glucoside, and luteolin are viewed as the key flavonoids in HBJ samples (Gong et al., 2021). Chen et al. (2020) demonstrated that SE pretreatment can cleave the hydrogen and glycosidic bands with mechanical shearing force (Chen et al., 2020). In this study, the contents of these flavone glycosides in the HBJ samples increased with the decrease of particle sizes because the conversion speed of the phenolic substances to these flavone glycosides was faster than the degradation speed of flavone glycosides. Besides, the continuous content decrease of acacetin was attributed to the hydrolysis of acacetin during SE pretreatment. The result revealed that the extraction yield of flavonoid glycosides and aglycones in 60‐mesh HBJ samples was the highest, which further demonstrated that the optimal sieve fractions for bioactive compound retention may be of intermediate size.

It was the first time to report on the changes in the total phenolic compound contents of HBJ samples with various sieve fractions during in vitro digestion. Phenolic compounds are more stable in acidic conditions. Besides, the increase of some phenolic compounds after the gastric phase of in vitro digestion is also associated with the release of phenolic compounds bound to the cell walls in the vegetal matrix (de Morais et al., 2020). Chen et al. (2015) reported that the antioxidant capacity became more reactive at acidic pH in the gastric phase and less reactive in the intestinal phase due to the transformation of a proportion of phenolic compounds into various structural forms with different chemical properties (Chen et al., 2015). Lingua et al. (2018) have found that the changes in chemical composition promoted by the digestive environment and the effect of digestive enzymes on carbohydrates and proteins may facilitate the release of flavanones (Lingua et al., 2018). In this study, the differences observed for the corresponding derived fractions could be associated with deprotonation of the hydroxyl moieties present on the aromatic rings of phenolic compounds because of the transition from an acidic to the alkaline environment during in vitro digestion.

## 5 CONCLUSION

In conclusion, the aim of this study was to investigate the effect of SE pretreatment on the changes in composition and in vitro digestion property of phenolics in the HBJ powder with various sieve fractions. The morphological structure and color attributes of the samples were altered by the SE pretreatment. The total content of phenolic compounds was strongly dependent on particle sizes, indicating a significant differential distribution of the compounds in the samples with various sieve fractions after SE pretreatment. The higher extraction yield of phenolic compounds was obtained in 60‐mesh samples. During in vitro digestion, phenolic compounds were more stable in acidic conditions. Based on PCA and PLS‐DA, three flavone glycosides (apigenin‐7‐O‐glucoside, luteolin‐7‐O‐glucoside, and linarin) were considered as the potential compounds responsible for the difference among three samples. The results demonstrated that SE pretreatment plays an important role in improving the extraction yield of phenolic compounds and digestion properties of HBJ powders with intermediate sieve fraction.

## CONFLICT OF INTEREST

The authors declare that there is no conflict of interest.

## ETHICAL APPROVAL

This article does not contain any studies with human participants by any of the authors.

## Supporting information

App S1

## Data Availability

The data that support the findings of this study are available on request from the corresponding author. The data are not publicly available due to privacy or ethical restrictions.
