# Supplementary material for: Effect of steam explosion pretreatment on the composition and bioactive characteristic of phenolic compounds in Chrysanthemum morifolium Ramat cv. Hangbaiju powder with various sieve fractions
Source: Food Sci Nutr. 2022 Mar 15;10(6):1888–98. doi: 10.1002/fsn3.2805 (PMC9179122; doi:10.1002/fsn3.2805)
Supplement: Supplementary file 1 — App S1 [file FSN3-10-1888-s001.docx]

**Appendix S1**

**SUPPLEMENTARY MATERIALS**

**Effect of steam explosion pretreatment on the composition and bioactive characteristic of phenolic compounds in *Chrysanthemum morifolium* Ramat cv. Hangbaiju** **powder with various sieve fractions**

**Tables**

1. Gradient conditions of HPLC.

**Figures**

Figure S1. Effect of SE pretreatment on total phenolic compounds in HBJ samples with various sieve fractions (20, 40, 60, 80, and 100 mesh), respectively. Different letters indicated a significant difference (*p* < 0.05).

Figure S2. The changes in total phenols and total flavonoids contents of HBJ samples with various sieve fractions (20, 40, and 60 mesh) before and after SE pretreatment during *in vitro* digestion. Different letters indicated a significant difference (*p* < 0.05).

Table S1 Gradient conditions of HPLC

| Time (min) | Mobile phase A (%) | Mobile phase B (%) |
| --- | --- | --- |
| 0 − 11 | 10 − 18 | 90 − 82 |
| 11 − 32 | 18 | 82 |
| 32 − 40 | 18 − 30 | 82 − 70 |
| 40 − 48 | 30 − 35 | 70 − 65 |
| 48 − 50 | 35 − 40 | 65 − 60 |
| 50 − 55 | 40 | 60 |
| 55 − 60 | 40 − 70 | 60 − 30 |
| 60 − 70 | 70 − 10 | 30 − 90 |

| 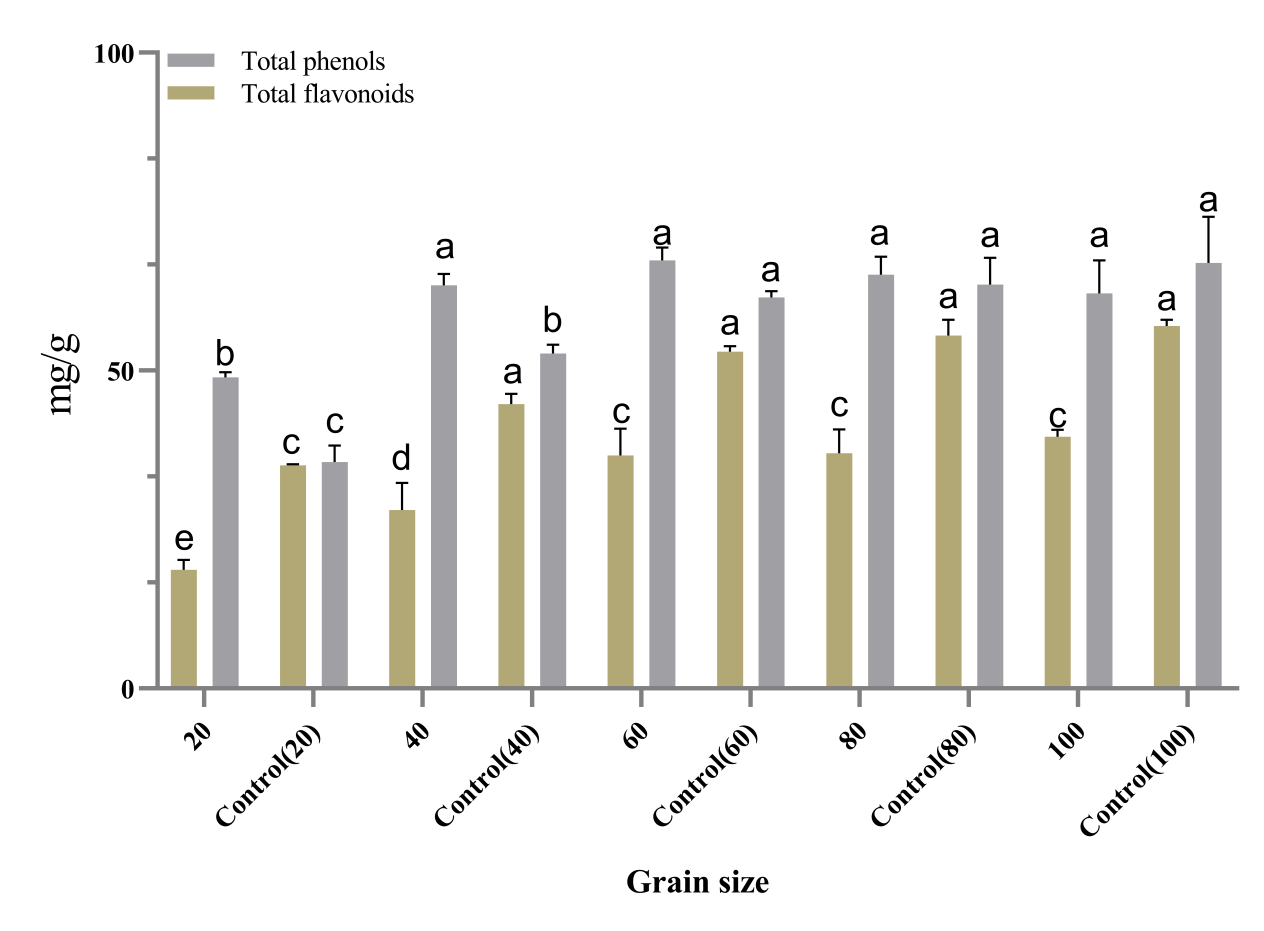 |
| --- |
| Fgure S1 |

| 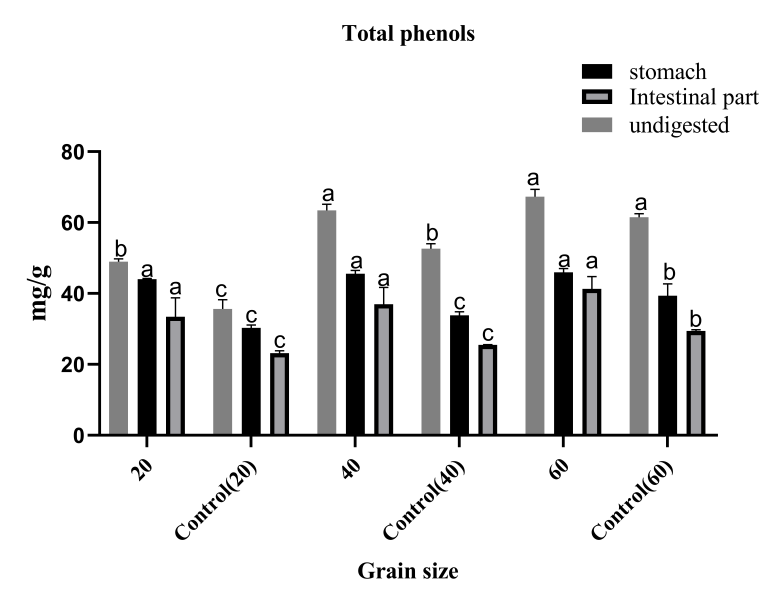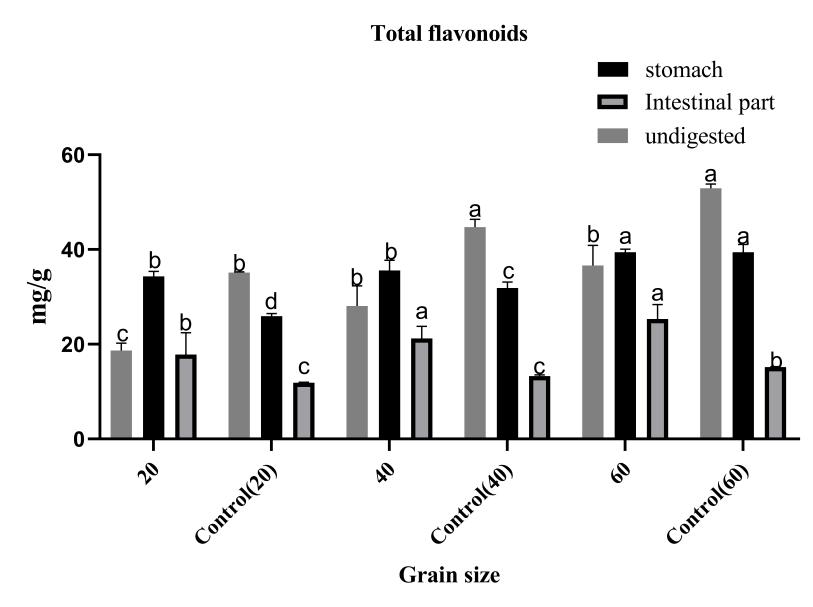 (A)  (B) |
| --- |
| Figure S2 |
